# Books Review

**Published:** 1870-10

**Authors:** 


### Books Review.

*The Practice of Medicine.* By THOMAS HAWKES TANNER, M. D., F. L. S. *Fifth American from the Sixth London Edition. Enlarged and Thoroughly Revised.* LINDSAY & BLAKISTON: Philadelphia. 1870.

We are happy to announce the appearance of the Fifth American Edition of this most complete and valuable work upon the Theory and Practice of Medicine. Tanner's Practice of Medicine is now one of the standard works in this country, upon practical medicine, as well as in Europe; and each new edition enlarges and improves it, bringing it up with the constant advances which are being made in our knowledge of and modes of treating disease. It can hardly be necessary to speak in detail of a book which, in some of its editions, is in nearly every physician's library; it only remains for us to say, that the last edition is the most full, comprehensive and complete, fully representing the most recent views in pathology and therapeutics, and entitled to the highest rank as a complete work upon Practical Medicine.

---

*Theory and Practice of Obstetrics.* By WILLIAM H. BYFORD, A. M., M. D. WILLIAM WOOD & CO.: New York. 1870.

Byford's Obstetrics affords the Student and Practitioner the science and practice of the art in the most available and reliable form. It is complete, though not large; it is full and perfect, and still is comprised in comparatively small space. It contains what is known, and commends itself to the profession, and especially to medical students, by its plain, well-considered complete teaching. Everything that can be said in favor of any work on this subject can be said of it; and to this you may add that it is compressed to small compass, and beautifully bound. Students in our Colleges cannot but select it as best adapted to their wants; it is a book we cannot too highly commend.

---

*Basham on Renal Diseases. A Clinical Guide to their Diagnosis and Treatment.*

Those members of the profession who are turning their atttention to the careful diagnosis of renal diseases, will be deeply interested in this work. It contains full directions for the most careful examination, both microscopic and chemical, of the urine; combining with it the general and clinical evidences present. We think it well adapted to the objects in view, viz.: "of promoting a practical and clinical knowledge of a class of diseases which are not without their difficulties in diagnosis, and of assisting both student and practitioner in their clinical observations." As a practical guide in the treatment of the various renal diseases it will prove valuable, the general subject being presented with the skill and observation of a clinical teacher rather than from a purely chemical or microscopic observation.

---

*Physicians Visiting List for* 1871.

We have received from Lindsay & Blakiston their "Visiting List" for 1871. It has the usual table of contents and the unexceptional style of former issues. It is printed on fine paper, is substantially bound, and is in every respect a most complete "visiting list," comprising all the advantages these pocket companions can possibly contain.

